# Data generated by the hybridization of mechanical properties of composite reinforced by piassava fiber fabric

**DOI:** 10.1016/j.dib.2018.11.084

**Published:** 2018-11-20

**Authors:** Genilson Cunha de Oliveira Filho, Rui Carlos de Sousa Mota, Ana Claudia Rangel da Conceição, Mirtania Antunes Leão, Oscar Olimpio de Araujo Filho

**Affiliations:** a UFPE, Av. Prof. Moraes Rego, 1235 – University City, CEP 50670-901 Recife, PE, Brazil; b IFBA, Campus Simões Filho, University street S/N Pitanguinha, CEP 43700-000 Simões Filho, BA, Brazil; c UFBA - Polytechnic School of the Federal University of Bahia, Street Aristides Novis, 2 – 8° Floor Federação, CEP 40210-630 Salvador, Bahia, Brazil; d IFBA, Emídio dos Santos street, S/N – Barbalho, CEP 40301-015 Salvador, BA, Brazil

## Abstract

Piassava (*Attalea funifera*), a palm tree endemic to Bahia, has a very flexible and resistant fiber. This data article aims to assess the effect of piassava fibers in polyester matrix on the mechanical properties of composites. Two composites were manufactured, one of the piassava fibers in polyester matrix and the other a hybrid of piassava and E-glass fibers in polyester matrix, by hot compression molding process, on which uniaxial tensile tests and three-point bending tests were later performed. The results so obtained show the effective contribution of piassava fibers as reinforcement in polymeric composites. This data article is related to “Effects of hybridization on the mechanical properties of composites reinforced by piassava fibers tissue” (de Oliveira Filho et al., 2018).

**Specifications table**

**Table:** 

Subject area	*Mechanical Engineering, Material Science*
More specific subject area	*Physical and mechanical properties of composites with fibers and hybridization*
Type of data	*Table, image (SEM), graph of Stress X Strain, graph of Stress X Flexural Strain, figures*
How data were acquired	*SEM, Machine Uniaxial tensile, Q150R ES, EMIC DL 30000.*
Data format	*Raw and analyzed data*
Experimental factors	*The alkali treatment was carried out by pre-washing in tap water and subsequent treatment with a solution of 10% NaOH concentration for at least one hour. After the mercerization, the fibers must be washed in distilled water for total removal of the NaOH solution as shown the*Fig. 1*and subsequently dried in an oven for 24 hours at a temperature of 60 °C for fabric preparation.*
Experimental features	*Stress-controlled uniaxial tensile tests and three-point bending tests in a universal testing machine.*
Data source location	*Materials Laboratory of the Institute Federal da Bahia (IFBA), Salvador City (Latitude: -12.9704, Longitude: -38.5124 12° 58′ 13″ Sul, 38° 30′ 45″ Oeste), Bahia, Brazil*
Data accessibility	*Data are available within the article*
Related research article	Genilson Cunha de Oliveira Filho**,** Rui Carlos de Sousa Mota, Ana Claudia Rangel da Conceição, Mirtania Antunes Leão and Oscar Olimpio de Araujo Filho, “Effects of hybridization on the mechanical properties of composites reinforced by piassava fibers” doi:10.1016/j.compositesb.2018.10.050[1] URL:https://doi.org/10.1016/j.compositesb.2018.10.050

**Value of the data**

• The data are useful for the development of composites that contemplate something in the minimum of these results before the mechanical properties studied.
• The data are useful for initial stage of development of composites before their mechanical properties can be studied.
• The data may be analyzed and compared with the data on composites of other fibers with which the piassava fibers may be hybridized.
• The data may guide other combinations of fibers and elements that may enhance other properties in the composites.

## 1 Data

**Fig. 1:**
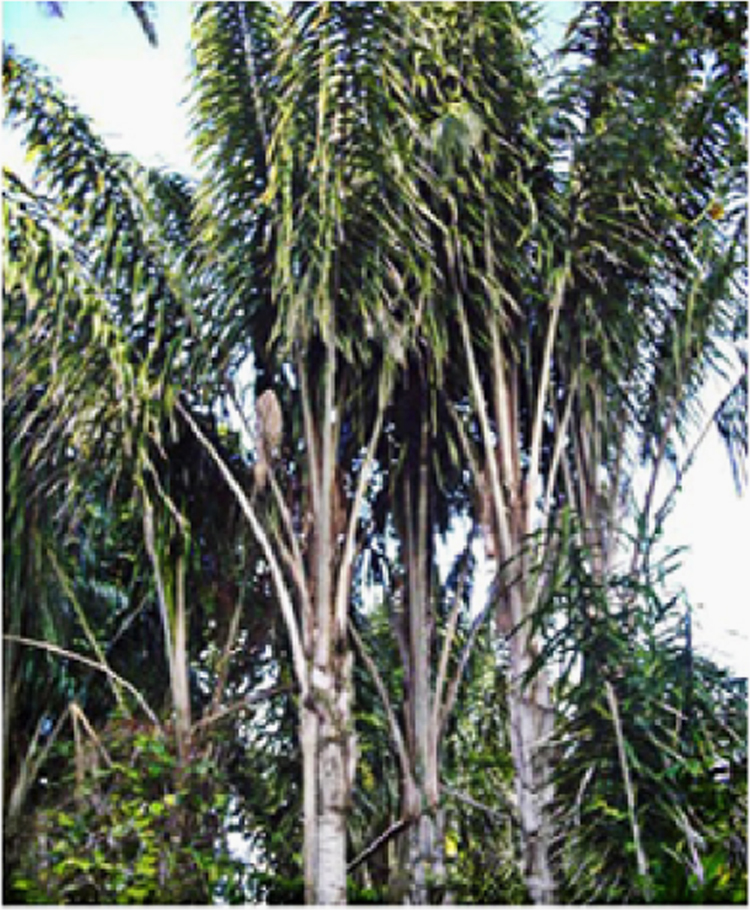
Piassava tree.

The piassava fiber (Attalea Funifera Mart), seen in Fig. 1 as in nature, used in preparation of laminated composite reinforced by piassava fiber fabric. It comprises data on mechanical properties (Table 1) and chemical composition (Table 2) of the piassava fiber.

**Table 1:** Mechanical properties of fibers.

*Fiber*	*Density*ρ (g/cm3)	*Tensile strength*σ (MPa)	*E* (GPa)	*References*
*Piassava*	*1.10–1.45*	*109–1750*	*5–6*	[2], [3]

**Table 2:** Chemical composition of main vegetal fiber.

*Fiber*	*Cellulose* (%)	*Hemicellulose* (%)	*Lignin* (%)	*Total fibers*	*Reference*
*Piassava*	*31.6*	*10.5*	*48.4*	*88.3*	[2]

**Fig. 2:**
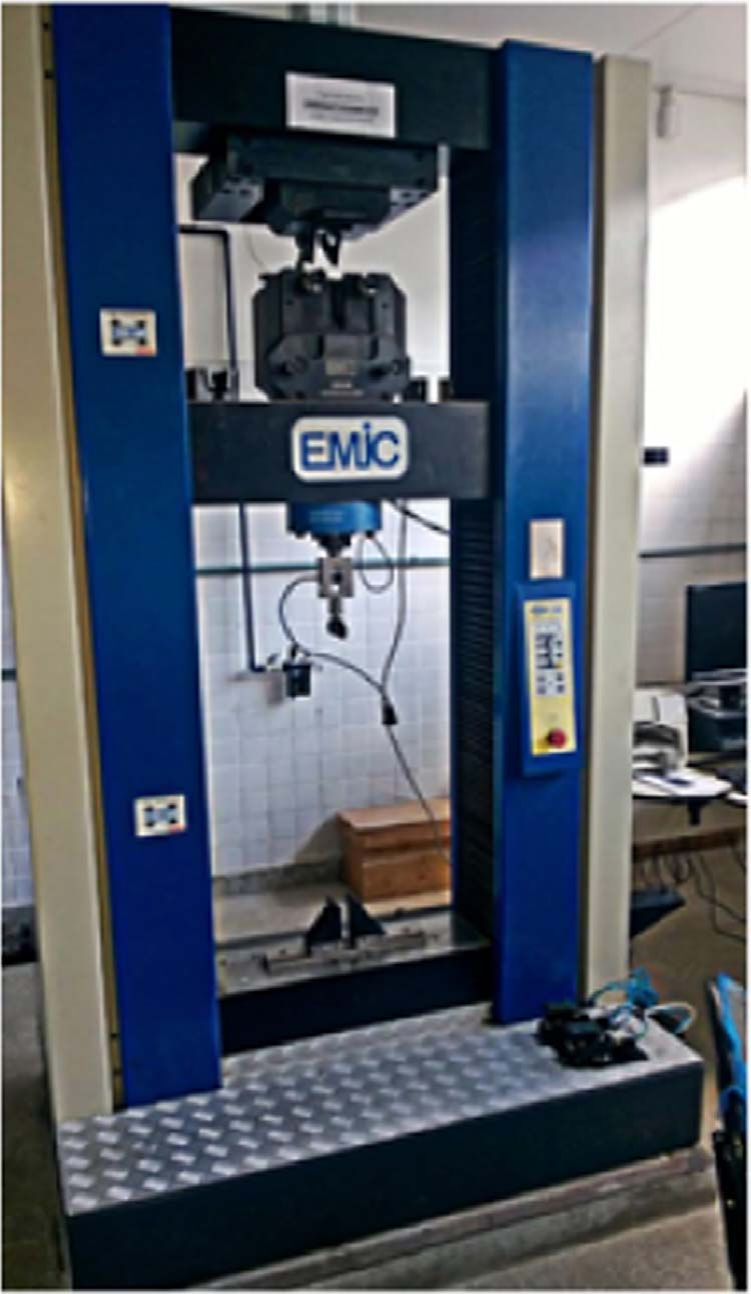
EMIC DL300KN.

Mechanical properties data refer to stress–strain diagrams obtained in stress-controlled uniaxial tensile tests and three-point bending tests with velocity of 1 mm/min by EMIC DL300kN equipment shown in Fig. 2.

Mechanical properties data refer to stress–strain diagrams obtained in stress-controlled uniaxial tensile tests and three-point bending tests with velocity of 1 mm/min by EMIC DL30000 equipment shown in Fig. 2.

## 2 Experimental design, materials and methods

The choice of piassava fibers as reinforcement is justified by their good specific properties and natural origin. However, as natural fibers have more physical variability and lower resistance compared to glass fibers, the option here was to make a hybrid composite of bidirectional E-glass fiber tissue and unidirectional piassava tissue manufactured in manual loom, in order to find the minimum loss in mechanical resistance.

**Fig. 3:**
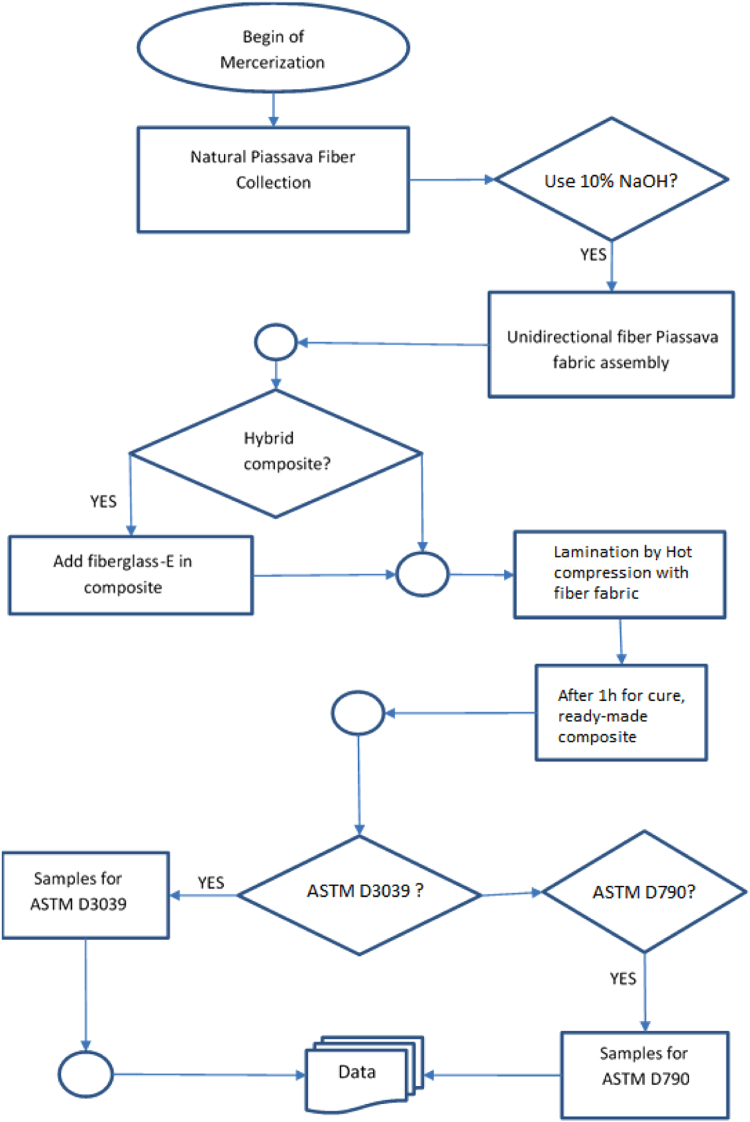
Lamination composite production flowchart.

The flowchart in Fig. 3 depicts the experimental procedure carried out in the work here reported.

The piassava fibers were first washed in distilled water for removal of earth and dirtiness and then subjected to alkali processing for reducing fiber polarity in relation to the apolar matrix. Afterwards, the fibers were placed in 10% NaOH solution for one hour and then were rinsed in distilled water and dried at environment temperature (25 °C) for 24 h.

The piassava reinforcing tissues were fabricated in a manual loom using the warp and weft technique in order to produce unidirectional tissues.

E-glass fiber and piassava fiber tissues, impregnated with orthophthalic unsaturated polyester resin and catalyzed at 1% (MEKP) were used as reinforcement in two different composites, a simple piassava fiber tissue laminate and a hybrid E-glass fiber bidirectional tissue with piassava tissue. Both glass-piassava hybrid and piassava composite laminates were manufactured by hot compression molding.

All tests were made in EMIC universal test equipment, with maximum 300 kN capacity (Fig. 2). The uniaxial tensile tests and three-point bending tests were performed in accordance respectively ASTM D3039 (2009) and ASTM D790 (2008) standards.

**Fig. 4:**
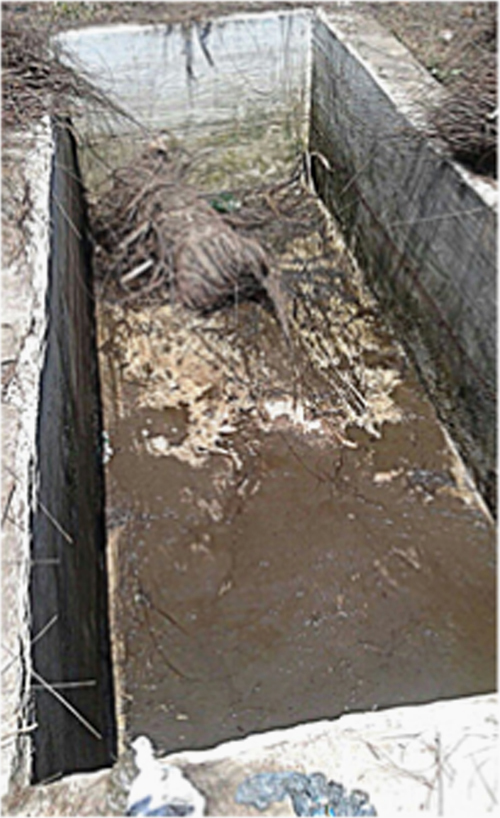
Washing piassava fiber in water.

**Fig. 5:**
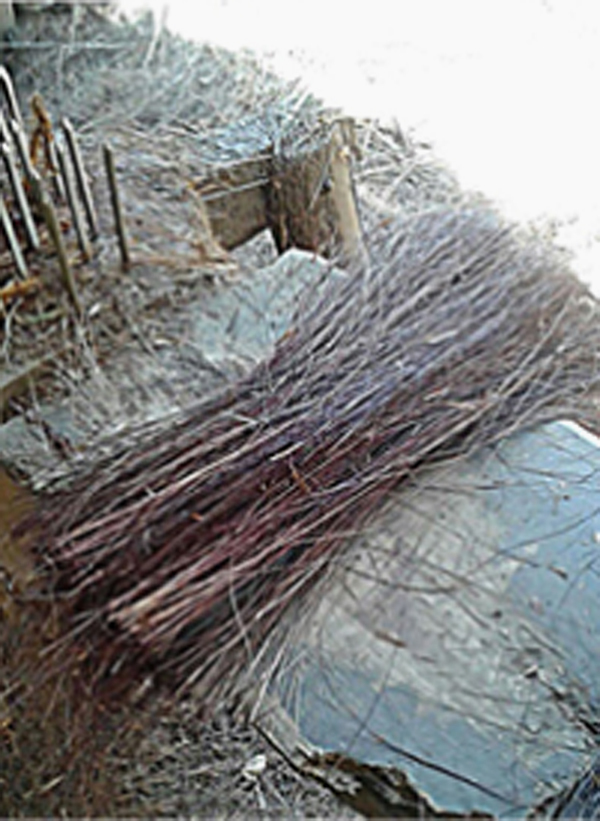
Cutting piassava.

Figs. 4 and 5 show initial procedures of collection and pretreatment of piassava fibers.

**Fig. 6:**
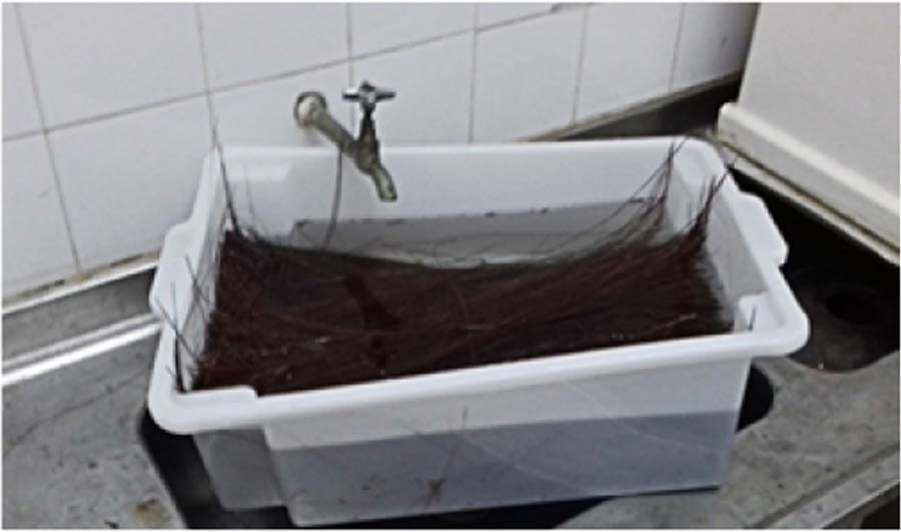
Mercerization.

**Fig. 7:**
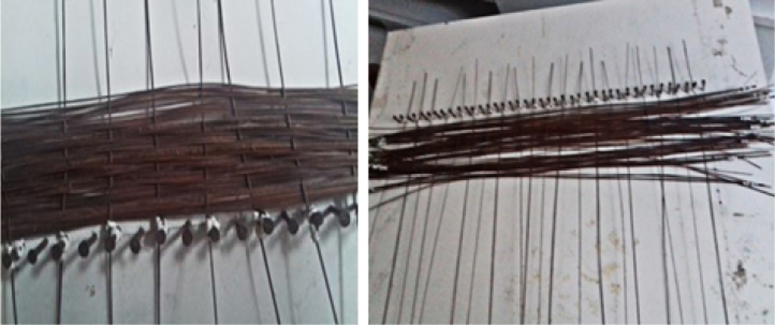
Assembling the piassava fibers for unidirectional fabric production.

Afterwards, the fibers are washed with 10% NaOH solution, as shown in Fig. 6, and after drying the unidirectional piassava fiber fabric shown in Fig. 7 is assembled.

**Fig. 8:**
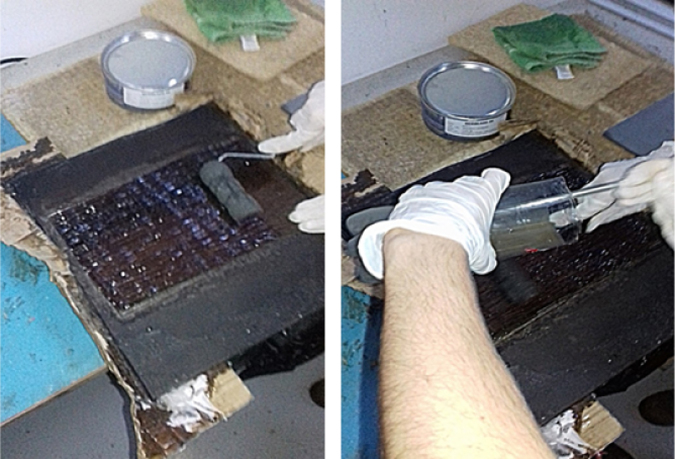
Process lamination.

After fabric production, resin is added, and the layered composite is formed, as Fig. 8 shows.

**Fig. 9:**
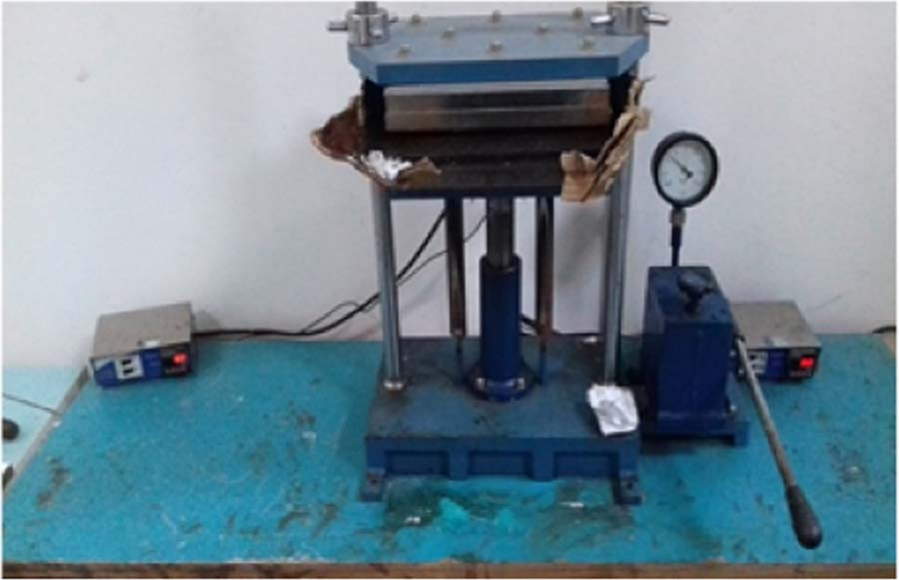
Compression molding.

**Fig. 10:**
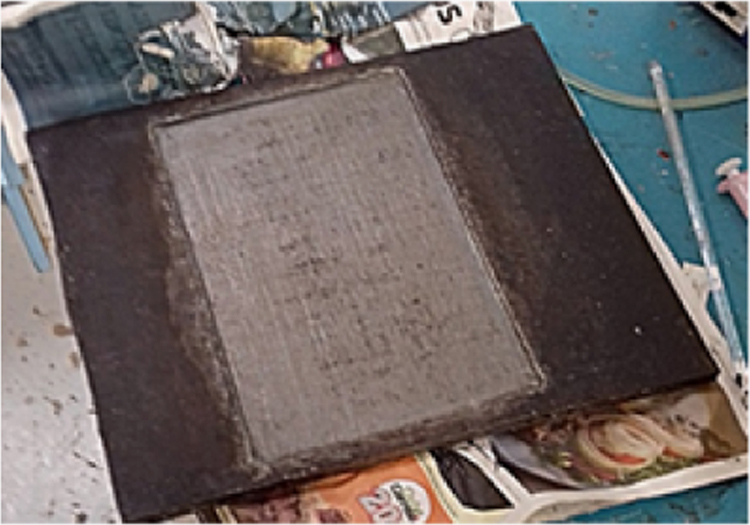
Composite laminate.

Composite manufacture was made by hot compression molding, Fig. 9. After one hour cure inside the mold, the test specimens were cut in accordance with ASTM D 3039 and D790 standards [4], [5] and composite laminate shown in Fig. 10.

**Fig. 11:**
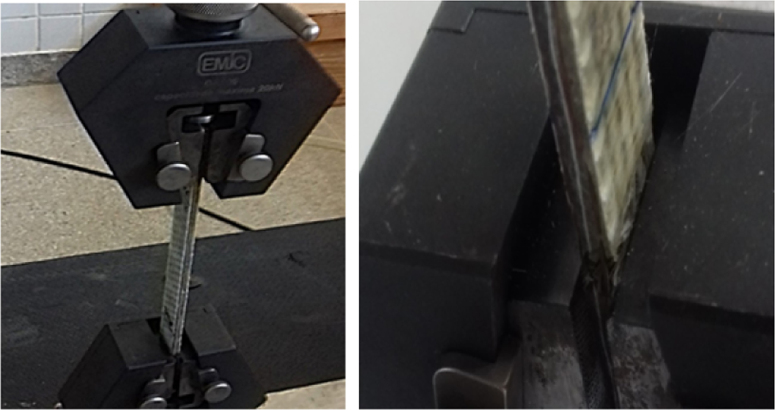
Example of sample in the test machine by ASTM D3039.

**Fig. 12:**
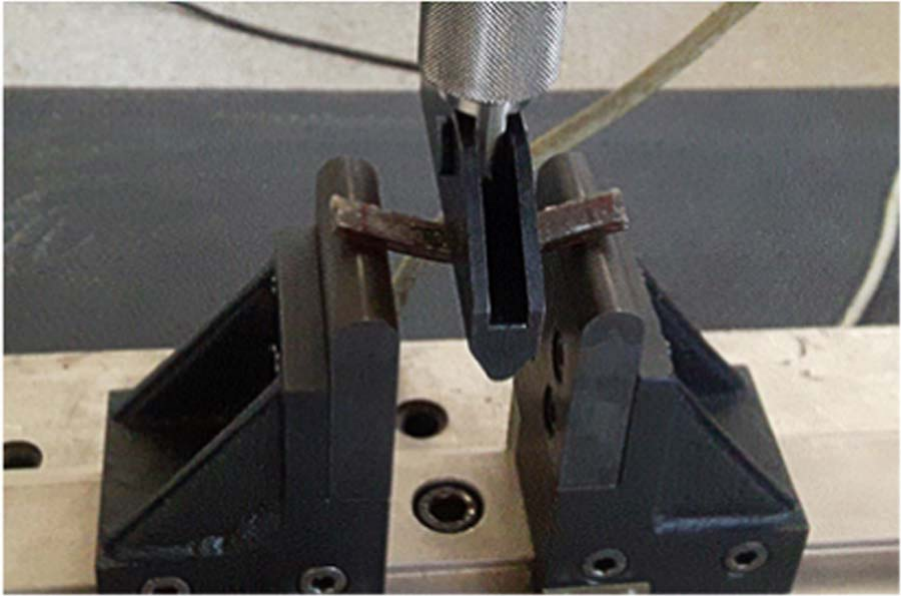
Example of test specimen in test equipment in accordance with ASTM D790.

Examples of test specimens mounted in test equipment, Figs. 11 and 12.
