# Supplementary material for: Data generated by the hybridization of mechanical properties of composite reinforced by piassava fiber fabric
Source: Data Brief. 2018 Nov 20;21:2330–8. doi: 10.1016/j.dib.2018.11.084 (PMC6280552; doi:10.1016/j.dib.2018.11.084)
Supplement: Supplementary file 1 — Supplementary material [file mmc1.docx]

**Conflicts of Interest Statement**

Manuscript title: “Data generated by the hybridization of mechanical properties of composite reinforced by piassava fiber fabric”

The authors whose names are listed immediately below certify that they have NO affiliations with or involvement in any organization or entity with any financial interest (such as honoraria; educational grants; participation in speakers’ bureaus; membership, employment, consultancies, stock ownership, or other equity interest; and expert testimony or patent-licensing arrangements), or non-financial interest (such as personal or professional relationships, affiliations, knowledge or beliefs) in the subject matter or materials discussed in this manuscript.

Author names: Genilson Cunha de Oliveira Filho, Rui Carlos de Sousa Mota, Ana Claudia Rangel da Conceição , Mirtania Antunes Leão and Oscar Olimpio de Araujo Filho
